# The effectiveness of Tai Chi on the depressive symptom of young adults with subthreshold depression: a study protocol for a randomized controlled trial

**DOI:** 10.1186/s13063-021-05054-6

**Published:** 2021-01-30

**Authors:** Xiaoting Xie, Jian Song, Jingfang Zhu, Mengyu Han, Youze He, Jia Huang, Jing Tao, Jingsong Wu

**Affiliations:** 1 Fujian University of Traditional Chinese Medicine, Fuzhou, 350122 China; 2 Key Laboratory of Orthopedics & Traumatology of Traditional Chinese Medicine and Rehabilitation (Fu Jian university of TCM), Ministry of Education, Fuzhou, 350122 China; 3 Fujian Collaborative Innovation Center for Rehabilitation Technology, Fuzhou, 350122 China; 4 TCM Rehabilitation Research Center Of SATCM, Fuzhou, 350122 China

**Keywords:** Tai Chi, Subthreshold depression, Young adults, HPA, Randomized controlled trial

## Abstract

**Background:**

Depression is seriously affecting the physical and mental health of young people worldwide. Subthreshold depression, as an early stage of depression, is essential for early prevention and treatment of depression. Tai Chi, as a traditional Chinese mind-body therapy, may become an alternative intervention. However, the neurophysiological mechanism of Tai Chi for young people with subthreshold depression remains unclear, restricting its further promotion and application. Therefore, rigorous randomized clinical trials are needed to further observe the intervention effect of Tai Chi on young adults with subthreshold depression and explore the neurophysiological mechanism.

**Method/design:**

This report describes a two-arm, randomized, parallel controlled trial with allocation concealment and assessor blinding. A total of 64 eligible participants are randomly allocated to the Tai Chi group and the waiting list group in a 1:1 ratio. Participants in the Tai Chi group receive 12 weeks of Tai Chi training, with a total of 36 times and each for 60 min. Specifically, the participants in the waiting list group are requested to maintain their routine lifestyle. In this study, the primary outcome measure is the mean change in scores on the PHQ-9 and HAMD-17 between baseline and 12 weeks; the secondary outcomes are the mean change in the scores on CES-D, CPSS, GAD-7, and PSQI. Besides, the saliva cortisol levels and *f*MRI are monitored to explore the mechanism of action of Tai Chi on subthreshold depression.

**Discussion:**

The protocol uses a randomized controlled trial to examine the effectiveness of Tai Chi for young adults with subthreshold depression and explore neurophysiological mechanisms. If the test results are positive, it can be verified that Tai Chi can promote the physical and mental health of young adults with subthreshold depression.

**Trial registration:**

Chinese Clinical Trial Registry ChiCTR1900028289. Registered on 17 December 2019

## Background

Major depressive disorder (MDD) is already a major burden of disease worldwide and is the second most psychiatric disease in the world that causes disability [1, 2]. Subthreshold depression, a pre-stage of MDD [3, 4], has been considered to be an important period of early intervention of MDD. Subthreshold depression is defined as a symptom of depression that not meets diagnostic criteria but also reduces the quality of life and function [5, 6]. It is the leading cause of disability in youth [7, 8], related to stress [9], anxiety [5], insomnia [10], drug abuse [11], social dysfunction [12], and even leads to suicide [13]. Although there are many antidepressant drugs for treating depression, there are still many problems such as drug resistance and side effects. More and more researchers have paid attention to non-drug treatments of subthreshold depression such as mind-body therapy and complementary and alternative medicine.

Tai Chi is a kind of mind-body aerobic exercise, which has been verified to benefit for depressive symptoms [14] and related symptoms such as stress [15] and insomnia [16]. It contains a series of psychologically beneficial components such as meditation, breathing regulation, mental control, and physical exercise [17]. Many studies have applied Tai Chi alone or in combination with the treatment of depression. For example, Lavertsky et al. [18] combined antidepressant with Tai Chi to jointly intervene in elderly depressed patients with partial response to escitalopram. They revealed that patients who received Tai Chi training had better improvement in depression level, and the quality of life and cognition; besides, the benefits of Tai Chi as adjunctive intervention far exceed standard antidepressant treatment or adjuvant medication. Some researchers even use it alone to treat MDD. Yeung et al. [19] used a small sample of randomized controlled trials to demonstrate that Tai Chi is feasible and safe in the treatment of Chinese American depression patients, observing an improvement in the treatment response rate and remission rate in the Tai Chi intervention group. Further research proves that Chinese Americans with major depressive disorder underwent a 12-week Tai Chi training. Compared with the health education group and the waiting list group, the response rate and remission rate of the Tai Chi group both exceeded 50%, indicating that Tai Chi can effectively reduce the anxiety and depressive symptoms of depressive patients and can be used as an adjuvant treatment for patients with depression. Chou et al. [20] explored the effect of Tai Chi’s social support on Chinese elderly patients with major depression. The results illustrated that Tai Chi had a significant effect on depressive symptoms when controlling age, gender, and education. The intervention effect is not significant when social support was controlled. Therefore, it is speculated that social support may be part of the reason for Tai Chi treatment of depression symptoms. The practice of Tai Chi was essentially a social activity.

A recent study used mindfulness combined with simplified 24 short-form Tai Chi Chuan to treat subthreshold depression in adolescence [21]. In this study, Zhang et al. [22] demonstrated that the depression level of adolescents with subthreshold depression can be improved by 8-week mindfulness-based modified Tai Chi Chuan. However, it is not clear whether mindfulness or tai chi are an effective part of intervention for subthreshold depression.

Among the many physiological factors leading to subthreshold depression, dysfunction of the hypothalamic-pituitary-adrenal (HPA) axis is considered to be an important cause [23]. Some studies have discovered that patients with subthreshold depression have dysfunction of HPA axis [24]. The HPA axis is part of the neuroendocrine system that controls the body’s stress response and regulates mental and emotional activities, such as stress and depression. There is evidence that the activity of the glucocorticoid receptor (GR) is decreased when the body is depressed, causing a decrease in the negative feedback inhibition of the HPA axis and excessive activation of the HPA axis [25]. GR can penetrate the blood-brain barrier and bind to the GR receptors in the brain, influencing the structure and function of the brain [26]. Research has indicated that hippocampus, prefrontal cortex, and amygdala are the most abundant brain regions of GR receptors [27] and directly related to emotional problems such as depression and psychological stress [28]. Some studies have revealed that mild depression or subthreshold depression were associated with a decrease in gray matter volume in the frontal cortex, anterior cingulate gyrus, thalamus, supraorbital/temporal lobe, and suprafrontal gyrus [29–31]. Li et al. discovered that compared with the healthy control group, the gray matter volume of the bilateral globus pallidus and the central anterior gyrus decreased in the patients with subthreshold depression, and the gray matter volume of the left thalamus and the right anterior cingulate cortex/medial prefrontal cortex increased [32].

Previous studies have illustrated that Tai Chi training has a regulatory effect on the function of the HPA axis and depression-related brain regions [33–35]. Nedeljkovic et al. [36] investigated that a 12-week, twice-weekly, 60-min Tai Chi practice can significantly improve the stress and negative emotions of the practitioner compared to the waiting list and further reduce the HPA axis response to stress and the concentration of salivary cortisol. Tao et al. [37] concluded that 12-week Tai Chi practice increased the gray matter volume of the medial temporal lobe, putamen, and insula. After a 12-week Tai Chi practice, a significant decrease in resting-state functional connections exhibited between the dorsolateral prefrontal cortex and the left superior frontal gyrus and anterior cingulate cortex [38].

Above all, this study uses a randomized controlled trial design to observe the effects of 12-week traditional Tai Chi on young adults with subthreshold depression from the perspective of depression-related symptom, HPA axis functions, and neuroimaging. This research provides evidence-based medical for the further application of Tai Chi in subthreshold depression.

## Methods/design

### Study design

**Fig. 1:**
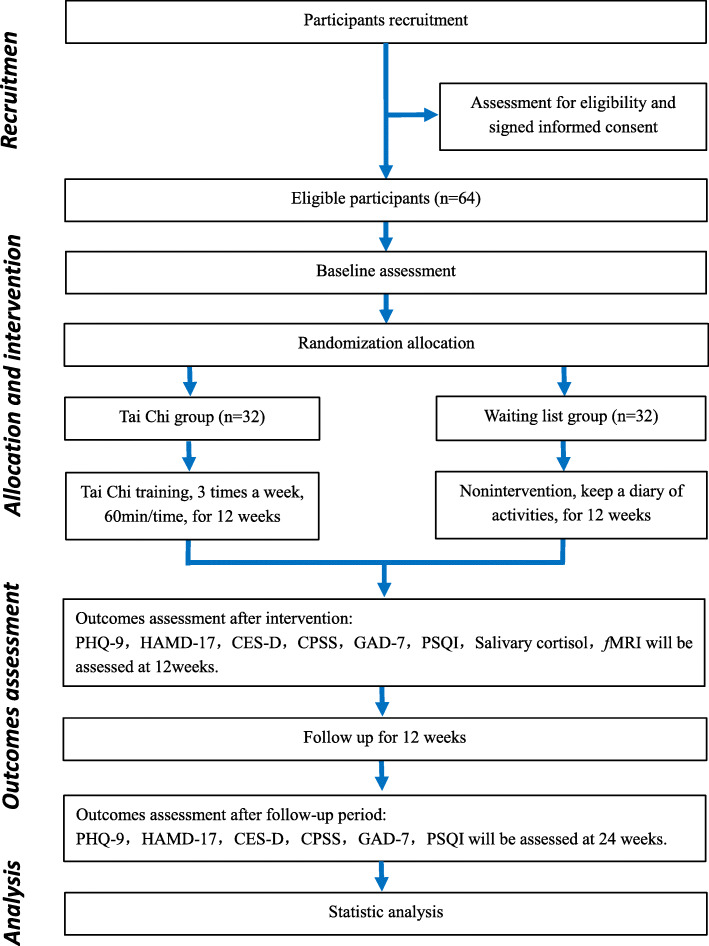
Flow diagram of participants. Abbreviations: PHQ-9, The 9-item Patient Health Questionnaire; HAMD-17, The 17-item Hamilton Depression Scale; CES-D, Center for Epidemiological Studies Depression Scale; CPSS, Chinese Perceived Stress Scale; GAD-7, A 7-item Generalized Anxiety Disorder; PSQI, Pittsburgh Sleep Quality Index; fMRI, functional magnetic resonance imaging

**Fig. 2:**
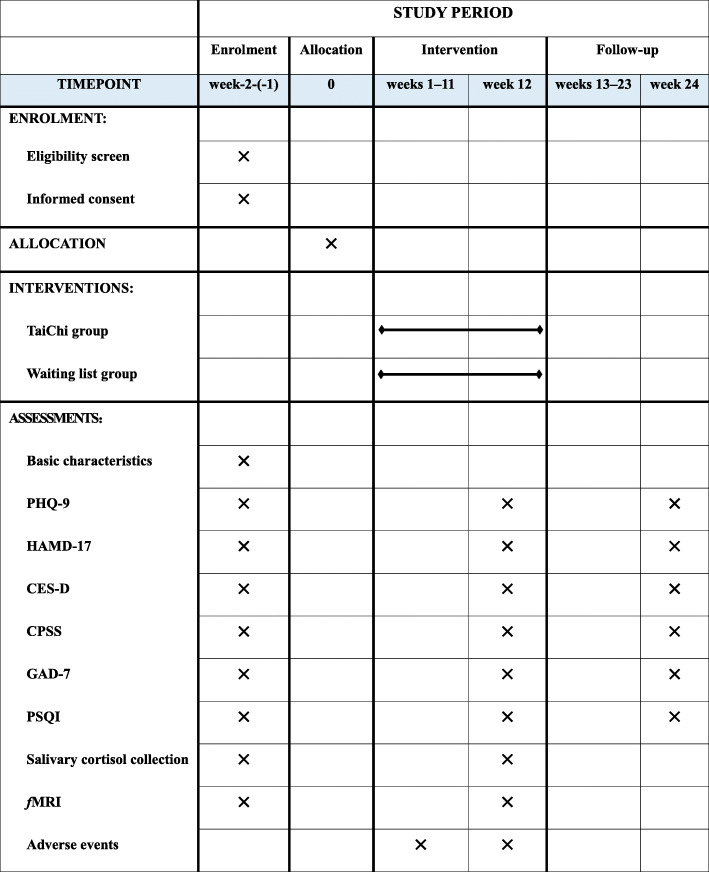
SPIRIT figure. Schedule Of enrolment, interventions, and assessments

The effectiveness of traditional Tai Chi training on young adults with subthreshold depression is assessed in a randomized, single-blind, and parallel-controlled trial. Outcome assessors and data analysts will be blinded. A total of 64 eligible participants are randomly allocated to the Tai Chi group and the waiting list group in a 1:1 ratio. Participants in the Tai Chi group receive 12 weeks of Tai Chi training, with three times a week, each for 60 min, a total of 36 times. The participants in the waiting list group are requested to maintain their routine lifestyle. Primary and secondary outcomes are measured at baseline, 12 weeks after the intervention, and after an additional 12-week follow-up period. Primary and secondary outcomes are measured at baseline, 12 weeks (at the end of intervention), and 24 weeks (after 12-week follow-up period). A flow diagram of the study design is illustrated in Fig. 1, and the time points for the study visits are presented in Fig. 2.

### Sample size estimation

In this study, the influence of Tai Chi on the depressive symptom of young adults with subthreshold depression was observed. When estimating the sample size, PHQ-9 and HAMD-17 were used as the main effect indicators to calculate the required sample size. Besides, the larger sample size was used as the sample size of this study.

The sample size estimation was based on the improvement in PHQ-9 (The 9-item Patient Health Questionnaire) scores. According to relevant published literature [22], the mean and its SD of PHQ-9 scores after the 8 weeks of mindfulness-based Tai Chi training for subthreshold depression adolescents were 6.03 and 2.18 points, respectively, while the mean and its SD of PHQ-9 scores in the control group were 8.10 and 2.52 points, respectively. According to the above results, a sample size of 58 participants was calculated to sufficiently detect the target effect size (0.879) with a type 1 error of 5% (*α* = 0.05) and 90% power (*β* = 0.10) by Gpower V.3.1.9.2 software. Assuming a dropout rate of 10%, a total of 64 participants are necessary, with 32 participants in each group.

Simultaneously, according to the research [21], the total score of HAMD-17 obtained by regular Tai Chi training to improve patients with major depressive disorders was 9 ± 5 points while the HAMD-17 score of control group patients with major depressive disorders was 15 ± 7 points. Based on the above results, a sample size of 46 participants was calculated to sufficiently detect the target effect size (0.986) with a type 1 error of 5% (*α* = 0.05) and 90% power (*β* = 0.10) by Gpower V.3.1.9.2 software. Assuming a dropout rate of 10%, a total of 52 participants are necessary, with 26 participants in each group.

Since the sample size calculated using PHQ-9 as the result indicator is more than HAMD-17, this study chose PHQ-9 as a basis to estimate the sample size of this study, and a total of 64 subjects were recruited.

### Participants and recruitment

A total of 64 participants were recruited at various communities in Fuzhou, China. Participants will be recruited by distributing leaflets, posters, recruitment information on WeChat, or other online platforms. Besides, a recruiting station in places where young adults are concentrated was set up, such as universities and business districts. Interested volunteers can contact the research assistants, who would screen applicants according to the inclusion criteria and exclusion criteria. The research assistant introduced the entire study and informed consent to the volunteers in detail, mainly including the research cycle and intervention methods. Meanwhile, the research assistant explains the grouping situation. Participants should obey random allocation and follow the prescribed scheme of each group (such as the participants had to agree not to participate in Tai Chi if they were randomized to the waiting list group); otherwise, they are excluded. Eligible volunteers are invited to join the study, sign informed consent, and then be scheduled for baseline assessment.

### Inclusion criteria

In order to be eligible for the study, participants must meet the following criteria needed:

1. Centre for Epidemiological Studies Depression Scale [CES-D] score ≥ 16, reaching the experience subthreshold depression [39].
2. The Mini International Neuropsychiatric Interview (MINI version 5.0) was used to determine that major depression was not achieved and there was no significant risk of suicide according to Diagnostic and Statistical Manual of Mental Disorders (Fifth Edition) (DSM-V) criteria.
3. Aged between 18 and 45 years.
4. No treatment related to depression has been received in the past 6 months.
5. Regularity mind-body exercises (Tai Chi, Baduanjin, Yoga, meditation, etc.) were not performed within half a year (regularity mind-body exercise refers to exercise at least twice a week, at least 20 min each time).
6. No contraindication of MR examination.
7. Written informed consent.

### Exclusion criteria

Exclusion criteria for participants were:

1. Meeting DSM-V criteria for a major depressive episode, bipolar disorder, or psychotic disorder.
2. Have a history of MDD in the past 6 months.
3. Recent bereavement.
4. In the use of glucocorticoid drugs, psychotropic substances or addiction disorders, such as drug dependence and alcohol abuse.
5. Suffering from endocrine and metabolic diseases (Cushing’s syndrome, Addison’s disease, etc.)
6. Have a history of head trauma or a history of serious diseases such as heart, liver, and kidney.
7. Pregnant women and lactating women.
8. Participate in other clinical trials that will affect the results of this study.

### Withdrawal or dropout criteria

Participants will withdraw from this trial according to the following:

1. Participants who decide to withdraw.
2. Development of serious diseases hinders the continuation of the trial.
3. Suffering from a serious adverse event (AE) related to the Tai Chi training.
4. Participations in the waiting list group have regularly engaged in Tai Chi exercise.

### Randomization and allocation concealment

After the baseline assessment, eligible participants were randomly assigned to the Tai Chi group and the waiting list group in a ratio of 1:1. The random allocation sequence was generated by an independent statistician using the PLAN procedure of the statistical software SAS 9.1. The random grouping sequence was hidden by opaque and sealed letters and managed by an independent research assistant who knows nothing about the recruitment, evaluation, and intervention of the participants. The eligible participants were informed of their allocation result by the independent research assistants via telephone.

### Blinding

In this study, it is impossible to blind the Tai Chi coaches and participants. However, statistician blindness and evaluator blindness were used in this study. Two levels of blindness would be used in this study: the first level of blindness with the letters “A” and “B” to represent the groups assigned by the participants, and the second level blind bottom with “A” and “B” to represent intervention measures, such as “Tai Chi group” and “waiting list group”. At the end of the trial, the data were entered into the database according to the participants’ codes. After the data were checked, the database was closed, and the first-level unblinding is performed to determine which group of participants were assigned to “A” and “B”. After completing all the data analysis, the second-level of unblinding is performed to determine whether the subjects were assigned to the Tai Chi group or the waiting list group.

### Intervention

#### Tai Chi group

The Tai Chi adopted in this study is the 24 forms of simplified Tai Chi training, which are recommended by the General Administration of Sport of China as a popular health sport [40]. Qualified coaches who have been teaching Tai Chi for more than 5 years will teach participants the correct Tai Chi training throughout the intervention period. In addition, all the coaches completed the required research and human protection training before starting the intervention course.

Participants in the Tai Chi group will receive 12 weeks of concentrated Tai Chi training, three times a week, a total of 36 times, each for 60 min including 10-min warmups, 45 min Tai Chi training, and 5 min cool down. The training will be held in the gymnasium of Fujian University of Traditional Chinese Medicine or Community activity center or park.

#### Waiting list group

The participants in the waiting list group will be requested to maintain their routine lifestyle and did not take any intervention measures. After the study (24 weeks), participants in the waiting list can choose to participate in Tai Chi training or accept other interventions.

All participants will be informed to record their daily activities or exercise information during the study to observe any effects of normal physical activity.

### Follow-up period

After 12 weeks of intervention, all participants will begin an additional 12 weeks of unsupervised follow-up period. The participant will resume their routine lifestyle during the follow-up period. After 12-week follow-up period, primary and secondary outcomes will be remeasured.

### Outcome measurement

The variables of this trial include basic characteristics, primary outcomes, secondary outcomes, and exploratory outcomes. The basic characteristics were measured at baseline (1–2 weeks before randomized) via a questionnaire. Primary and secondary outcomes were measured at baseline, the end of the intervention (12 weeks after randomization), and the end of the follow-up period (24 weeks after randomization). Exploratory outcomes were measured at baseline and the end of the intervention period (12 weeks after randomization). All outcomes are evaluated by the experienced assessors who do not know the allocation results of participants.

#### Primary outcome measures

The primary outcome measure changes in the PHQ-9 and HAMD-17 between baseline and 12 weeks.

The 9-item Patient Health Questionnaire (PHQ-9) is a self-report measure used to assess 9 depressive symptoms according to the DSM-IV depression criteria. It has a total of 9 items, each with a score of 4 points (0–3 points) and the total scores’ range of 0–27 points. This score can be used to describe the patient’s symptoms, divided into five categories: none (0–4), mild (5–9), moderate (10–14), moderately severe (15–19), and severe (20–27) [41]. PHQ-9 has been translated into Chinese, and its Chinese version has demonstrated reliability and validity [42].

The 17-item Hamilton Depression Scale (HAMD-17) [43] is the most widely used scale in the clinical evaluation of depression, including17 items that assess function in five areas: anxiety/somatization, retardation, cognitive impairment, sleep disturbance, and weight change. Higher scores indicate more severe depression.

#### Secondary outcomes measures

Center for Epidemiological Studies Depression Scale (CES-D) [44] designed to measure the frequency of depression-related symptoms in a subject over the past week. It is a 4-point ordinal scale: rarely or none of the time (< 1 day); some or a little of the time (1–2 days); occasionally or a moderate amount of the time (3–4 days); and most or all of the time (5–7 days). CES-D score was between 0 and 60, with higher scores indicating greater symptom burden. The cut-off score of CES-D was 16, indicating significant or mild depression [39].

Chinese Perceived Stress Scale (CPSS) [45] is applied to assess the stress, consisting of 14 items to reflect the uncontrollability and tension of stress. Each item ranges from 0 (never) to 4 (very often). Higher aggregate scores express greater perceived stress.

A 7-item Generalized Anxiety Disorder (GAD-7) [46] is a self-report scale designed to measure the frequency of anxiety symptoms in the past 2 weeks. It is composed seven items, and each item score ranges from 0 (not all) to 3 (almost daily); 5 points, 10 points, and 15 points represent thresholds for mild, moderate, and severe anxiety symptoms.

Pittsburgh Sleep Quality Index (PSQI) is used to measure the quality of sleep [47]. The Chinese version of PSQI has been reported acceptable internal consistency, test-retest reliability, construct validity, and criterion-related validity. The questionnaire has nineteen individual items used to generate seven composite scores. The results provided numbers in seven categories: subjective sleep quality, sleep latency, sleep duration, habitual sleep efficiency, sleep disturbances, use of sleeping medication, and daytime dysfunction. The sum of the seven component scores ranges from 0 to 21; higher scores indicate poorer subjective sleep quality [48].

#### Exploratory outcomes measures

Saliva cortisol levels assess to evaluate the functional status of the HPA axis (Cortisol Awakening Response (CAR) and nocturnal cortisol level). Saliva was collected using the Salivette sampling device (Sarstedt, Italy). The Salivette sampling device was distributed to the subjects before the collecting day. Besides, the subjects were instructed to ensure that they fully understand the methods and precautions of saliva collection. Subjects collected saliva at home or dormitory at 0, 30, 45, and 60 min after awakening and 23:00 before sleeping and were asked to avoid eating, drinking alcohol or coffee, brushing teeth, smoking, or exercising within 60 min after waking and 30 min before other saliva collections. Do not collect samples when oral diseases, inflammation, or lesions exist (blood contamination). The subjects would chew the polyester swab of the Salivette sampling device for at least 1 min and then put the polyester swab back into the inner tube of the Salivette sampling device. These devices were sent to the laboratory as soon as possible. Saliva samples recovered from a polyester swab by centrifugation at 3000 rpm for 15 min. Then, clear saliva was obtained in the outer tube and frozen at − 80 °C until analysis. Salivary cortisol concentrations were determined using a salivary cortisol ELISA kit (DRG Diagnostics, Germany). The inter-assay coefficient of variation was less than 13.6%, and intra-assay coefficient of variation was less than 6.1%, with a minimum detectable concentration of 0.09 ng/ml.

Structure and function of related brain regions were measured using functional magnetic resonance imaging (*f*MRI), which is a non-invasive method for examining brain activity and structure. T1-weighted structural images were acquired with the three-dimensional magnetization-prepared rapid acquisition gradient-echo (3DMPRAGE) sequence (sagittal scanning, TR/TE/FOV = 2000 ms/1.73 ms/240 mm × 240 mm, flip angle (FA) = 15 degrees, layers = 160, layer thickness = 1 mm, imaging matrix = 256 × 256). The resting-state sequence was performed (axial (nonoblique), TR/TE/FOV = 2000 ms/30 ms/220 mm × 220 mm, flip angle (FA) = 90 degrees, layers = 37, layers = 3.5 mm, imaging matrix = 64 × 64, time points 180). The fMRI scan was measured by professional operators at the Affiliated Rehabilitation Hospital of FJTCM using a 3.0-T signal MRI scanner (GE Healthcare, Little Chalfont, UK) with a birdcage head coil. MR scanning was performed at baseline and end of the intervention period (12 weeks after randomization).

### Safety evaluation

During the study intervention, any adverse events of the subject will be monitored, recorded in the adverse event case report form, reported to the research assistant, and analyzed for causality related to Tai Chi training and the severity of the adverse event.

During the intervention, any adverse events (defined as any functional impairment caused by the intervention, such as knee or ankle sprain, increased depression, knee pain, hypoglycemia) will be recorded on the case report form (CRF). If any adverse event occurs, the coaches or project managers will provide the corresponding treatment to the participant. Serious adverse events should be filled in a report form and reported to the Ethics Committee of Fujian University of Traditional Chinese Medicine Subsidiary Rehabilitation Hospital immediately.

Based on various factors, the safety evaluation of the subjects is conducted. The evaluation results are divided into level 1 (safe, without any adverse reactions); level 2 (safer, with mild adverse reactions, no need to do any treatment, subjects can continue to participate in training); level 3 (have safety problems, have moderate adverse reactions, and subjects can continue training after treatment); and level 4 (the study was discontinued due to serious adverse reactions) *(Common Terminology Criteria for Adverse Events (CTCAE) Version 5.0, US Department of Health and Human Services, 2017).*

### Data collection and management

The evaluator used a paper case report form (p-CRF) to collect data. The research assistant conducted quality control of the data collection and input this data into the Microsoft Office Excel (Microsoft, USA). All data entered into the Excel were imported into the data management platform (FJTCM yun) at http://10.252.47.2 and managed by an independent organization. All data were treated with the highest confidentiality.

When participants withdrew from a trial, the investigator asked the participant whether the participant was willing to participate in follow-up data collection; if the participant was willing, every effort would be made to obtain the follow-up data.

### Statistical analyses

Analysis of all data in this trial was performed by an independent statistician who is not involved in the evaluation of the results using the SPSS V.24.0 software package, with a statistical significance of a two-sided *p* value less than 0.05. Normally distributed continuous variables were described as mean ± standard deviation (SD), and non-normal variables were described as the median and interquartile range (IQR). Categorical variables were reported as frequencies or percentages. In this study, basic data including age, gender, BMI [49], education level [50], family factors [51], economic ability [52], and screen time [7] were collected, and basic data were proposed as covariates in this study. Analysis of the primary and secondary outcomes was performed based on the intention-to-treat (ITT) population and per-protocol (PP) population. The result of the ITT analysis was compared with that of the PP analysis to determine whether the results are consistent. Multiple imputations were used to impute missing data. ITT analysis was performed on all subjects in the Tai Chi group and waiting list group. In this study, after the subjects were randomly divided into groups, regardless of whether they received the treatment of the group, they were eventually included in the assigned group for statistical analysis, even though they withdrew from the trial due to adverse reactions. Differences between groups in the Tai Chi group and the waiting list group at each time point (12 weeks after intervention or 12 weeks’ follow-up) were analyzed using Student’s *t* test or Mann-Whitney *U* test. The interaction effect of group × time was analyzed by the linear mixed model with restricted maximum likelihood. Pearson correlation or Spearman correlation was adopted for correlation analysis.

### Quality control

Before the implementation of the project, we should draw up a standard research manual and case report form, hire neuropsychological experts to conduct standardized training for the research evaluators, and understand each step (method) of the project implementation in detail to ensure the quality of the research. The clinical trial quality control committee is composed of one professor, one associate professor, and one doctor in the project team. The researchers report the clinical trial progress to the clinical trial quality control committee for evaluation every month.

### Dissemination policy

Results will be published in peer-reviewed journals wherever possible, and the results will be communicated to each participant, health care professionals, and other relevant groups. All researchers and other colleagues involved in the future will become coauthors of this research based on their individual contributions.

## Discussion

It is widely believed in the academic community that practicing Tai Chi is of great benefit to promoting and maintaining physical and mental health [17]. Previous studies have demonstrated that Tai Chi may reduce depressive symptoms and related symptoms such as stress and insomnia in patients with depression [16, 21]. However, the treatment effect of traditional Tai Chi alone on subthreshold depression remains unclear.

In this experiment, a rigorous random, parallel control design was conducted to observe the effectiveness and safety of Tai Chi training on subthreshold depression patients. Furthermore, the physiological effect of the action of Tai Chi was further explored by observing the cortisol level (HPA axis function) and neuroimaging (*f*MRI). This study provides evidence that Tai Chi can treat subthreshold depression.

The control group was set as the waiting list group, and there may be some confounding factors, such as the Hawthorne effect and bad mood of the control group.

The “Hawthorne effect” refers to the effect of deliberately changing some behaviors or verbal expressions when people realize that they are being paid attention to or observed. First, in this study, the researchers maintained their attention and communication with the control group by recording weekly activity logs and regularly holding some entertainment activities to minimize the influence of confounding factors in the waiting list group on the research results. Second, in the evaluation of this study, subjective and objective outcome indicators (such as scales, MRI, and cortisol) were used to detect the study participants to reduce the bias of confounding factors.

To improve the quality of the research, some methods were employed in this study. Senior physical education teachers are arranged as Tai Chi training coaches to ensure the standard of the patient’s Tai Chi training. The participant in the Tai Chi group gathered at a fixed place and time to practice. To control the bias caused by the amount of exercise, all participants were required to record activity logs. Besides, Tai Chi training was supervised by two research assistants to guarantee that both the coaches and the participants practice carefully.

Cortisol has circadian rhythm and pulsation; the reliability of evaluating the functional status of the HPA axis only by collecting a single sample at a single time point was poor [53]. Our study collected salivary cortisol at multiple time points on the same day (0, 30, 45, and 60 min after awakening; 11 p.m.) to observe changes in the cortisol awakening response and nocturnal cortisol level associated with Tai Chi intervention.

Moreover, a return visit at 12 weeks after the end of the intervention was conducted to observe the long-term effects of Tai Chi training on young adults with subthreshold depression.

However, this trial may have some potential limitations. Firstly, it is difficult for people involved in RCT to become blind in a non-pharmacological trial [54]. Coaches and participants cannot be blinded in this study. Tai Chi coaches cannot be involved in recruiting, evaluating outcomes, or analyzing data in this study while performance discrepancies may be inevitable. Besides, this study used a randomized trial design and random assignment concealment. Random allocation is the responsibility of independent non-evaluators. Outcome assessors and statistical analysts were blinded to guarantee the test results authenticity and objectivity. Moreover, the project leader retained test quality control staff, and assessors and data analysts retained results until the end of the entire study period. Secondly, all participants came from the same city, contributing to reducing the sample representativeness.

To sum up, this study is the first randomized controlled trial to systematically evaluate the influence of traditional Tai Chi for patients with subthreshold depression from the perspective of subjective and objective indicators. If this trial exhibits significant results, a shred of rigorous evidence could be provided for the application of Tai Chi training among young adults with subthreshold depression, as well as a new way to prevent and reduce the global burden of MDD.

## Trial status

This protocol is the first version 1, which approved on 17 December 2019. The trial was started on 1 January 2019. We hope to achieve our research objectives by December 2021.

## Data Availability

To protect the privacy of participants, the datasets generated or analyzed during the current study have not been made publicly available, but they can be obtained from the corresponding authors if there are reasonable requirements.

## Abbreviations

MDD: Major depressive disorder
HPA: Hypothalamic-pituitary-adrenal
GR: Glucocorticoid receptor
PHQ-9: The 9-item Patient Health Questionnaire
DSM-IV: Diagnostic and Statistical Manual of Mental Disorders (Fourth Edition)
AE: Adverse event
HAMD-17: The 17-item Hamilton Depression Scale
CES-D: Center for Epidemiological Studies Depression Scale
CPSS: Chinese Perceived Stress Scale
GAD-7: A 7-item Generalized Anxiety Disorder
PSQI: Pittsburgh Sleep Quality Index
CAR: Cortisol Awakening Response
fMRI: Functional magnetic resonance imaging
p-CRF: Paper case report form
e-CRF: Electronic case report form
